# The Fifteenth International Congress of Medicine, Lisbon, 1906

**Published:** 1905-12-09

**Authors:** 


### THE FIFTEENTH INTERNATIONAL CONGRESS OF MEDICINE, LISBON, 1906.

A meeting of the National Committee for Great Britain and Ireland of the fifteenth International Congress of Medicine was held in the rooms of the Medical Society of London on November 30, 1905. Dr. Pavy, F.R.S., was in the chair, and amongst those present were Sir Dyce Duckworth, M.D., Dr. Ferrier, F.R.S., Dr. Radcliffe Crocker, Dr. Gordon Dill, Dr. Frew of Kilmarnock, Mr. W. H. H. Jessop, Mr. L. E. Creasy, Dr. Boyd Joll, and the Honorary

Secretaries, Mr. D'Arcy Power and Dr. Clive Riviere. The Secretaries made a report upon the travelling facilities offered to members going to the Lisbon Congress, which is to be held in Easter week next year. The arrangements with the railway companies are not yet complete, but a substantial reduction in fares is promised, though there will be the same troublesome formalities as were enforced at the Madrid Congress. Messrs. Thomas Cook and Son, acting in conjunction with Messrs. Anderson and Green of the Orient-Pacific Line, have chartered the *Ophir*, a twin-screw steamer, 6,814 tons register and 10,000 horse-power. The steamer will leave Tilbury at 2 P.M. on the Thursday before Good Friday, and will arrive at Tilbury again at 8 A.M. on Sunday, April 29. The boat will call at Cherbourg, Vigo, Tangier, Gibraltar and Oporto. She will lie at Lisbon for six days in order that her passengers may make the ship their home while attending the Congress and be spared the discomforts and risks of the shore hotels at a time when they are certain to be overcrowded. The usual routine of meals will be maintained on board, and regular communication with the shore will be kept up. During the stay of the *Ophir* at Lisbon a series of excursions will be made to suit those passengers whose time is not fully occupied at the Congress. The fare for the seventeen days' cruise (exclusive of shore excursions) will be from fifteen guineas upwards, according to the position of the cabin. The *Ophir*, it will be remembered, is the vessel which was commissioned as H.M.S. *Ophir*, and conveyed T.R.H. the Prince and Princess of Wales on their tour round the world. Early application for berths is desirable as a considerable number of the cabins are already engaged. Application should be made to Messrs. Thomas Cook and Son, Ludgate Circus, E.C., or to the Orient-Pacific Line, Fenchurch Avenue, E.C. A few berths are being reserved for delegates of the different scientific societies who are duly accredited to the Lisbon Congress.

The Travel Bureau, of 29 Cockspur Street, Charing Cross, S.W., has arranged two tours in connection with the International Congress at Lisbon. The first is under the leadership of Mr. J. B. Banks, F.R.G.S. The party will leave Charing Cross on Sunday, April 15, 1906, will sleep at Paris, and arrive in Lisbon at midnight on Tuesday, April 17. The return journey will commence on Friday, April 27, and will end in London on Wednesday, May 2. The inclusive first-class fare, with excursions to Cintra and Mont 'Estoril and two days at Busaco, is thirty-two guineas. This includes the *train de luxe* from Paris to Lisbon, but passengers who wish to use the Sud express on the way home must book their own seats and pay the excess fare. The Travel Bureau has also arranged for the conveyance of passengers by the Royal Mail steamer *Ambrose*, a vessel of 4,130 tons register, in the South American service. Passengers can join the R.M.S. *Ambrose* at Liverpool or at Havre. The fare—first class throughout—is eleven guineas from Liverpool back to Liverpool, or twelve guineas from London to London. Berths on the homeward voyage cannot be selected before leaving England, but a list is kept showing the order in which passengers are booked, and berths will be allotted on the returning steamer in accordance with this list.

The National Committee nominated a physician, a surgeon, and a general medical practitioner, persons of distinction, to serve as Presidents *d'honneur* at the Lisbon Congress, and considered a letter from Dr. F. G. Bushnell in regard to the formation of an International Health Ministry.

